# The Effect of Cultivation Practices on Agronomic Performance, Elemental Composition and Isotopic Signature of Spring Oat (*Avena sativa* L.)

**DOI:** 10.3390/plants11020169

**Published:** 2022-01-09

**Authors:** Aleš Kolmanič, Lovro Sinkovič, Marijan Nečemer, Nives Ogrinc, Vladimir Meglič

**Affiliations:** 1 Crop Science Department, Agricultural Institute of Slovenia, Hacquetova ulica 17, SI-1000 Ljubljana, Slovenia; lovro.sinkovic@kis.si (L.S.); vladimir.meglic@kis.si (V.M.); 2 Department of Low and Medium Energy Physics, Jožef Stefan Institute, Jamova 39, SI-1000 Ljubljana, Slovenia; marijan.necemer@ijs.si; 3 Department of Environmental Sciences, Jožef Stefan Institute, Jamova 39, SI-1000 Ljubljana, Slovenia; nives.ogrinc@ijs.si; 4 Jožef Stefan International Postgraduate School, Jamova Cesta 39, SI-1000 Ljubljana, Slovenia

**Keywords:** *Avena sativa* L., oat, cultivation practice, yield, energy dispersive X-ray fluorescence (EDXRF), elements, isotopic signature

## Abstract

The present study investigated the effects of cultivation practices on grain (oats) yield and yield components, such as straw yield, harvest index, thousand kernel weight, and plant lodging. In addition, multi-element composition and isotopic signature (*δ*^13^C, *δ*^15^N) of the oat grains were studied. The spring oat cultivar ‘Noni’ was grown in a long-term field experiment during 2015–2020, using three management practices: control without organic amendment, incorporation of manure every third year and incorporation of crop residues/cover crop in the rotation. Synthetic nitrogen (N) (0, 55, 110 and 165 kg/ha) was applied during oat development in each system. Multi-element analysis of mature grains from two consecutive years (2016 and 2017) was performed using EDXRF spectroscopy, while stable isotope ratios of carbon (C) and nitrogen (N) were obtained using an elemental analyzer coupled to an isotope ratio mass spectrometer (EA/IRMS). The results show how cultivation practices affect yield components and isotopic and elemental signatures. Increasing the N rate improved both the oat grain and straw yields and increased susceptibility to lodging. The results show how the elemental content (Si, Ca, Zn, Fe, Ti, Br and Rb) in the oat grains were influenced by intensification, and a noticeable decrease in elemental content at higher N rates was the result of a dilution effect of increased dry matter production. The mean *δ*^15^N values in oat grains ranged from 2.5‰ to 6.4‰ and decreased with increasing N rate, while *δ*^13^C values ranged from −29.9‰ to –28.9‰. Based on the *δ*^15^N values, it was possible to detect the addition of synthetic N above an N rate of 55 kg/ha, although it was impossible to differentiate between different management practices using stable isotopes.

## 1. Introduction

The oat (*Avena sativa* L.) is a minor, versatile whole-grain cereal used for food, animal feed, and non-food products [1]. Oats are grown worldwide and are seventh in global cereal production statistics after maize, wheat, rice, barley, sorghum and millet [2]. Nevertheless, the area planted with oats accounts for 1.4% of the total cereal area, while the production volume accounts for less than 1% of total cereal production [1,3]. The production potential of oats is generally lower than that of wheat or barley, probably due to lower genetic gains compared to other cereals, poorly optimized agronomic practices, and the prevalence of oats in low production environments, as they tolerate cool, moist, acidic and alkaline soils, and to some extent even saline soils [4]. In such environments, oat yields can rival those of other cereals, as shown by the example in the UK, where oat yields were comparable or higher to barley in the period 1996–2007, and yield increases were similar to wheat in the period 1985–2007, despite considerably less investment in oat breeding [5]. In high yielding environments, oat yields could not compete with those of wheat, barley and maize. However, it is estimated that oat yields have improved by 139% worldwide since the 1960s, although this improvement is smaller than for other major cereals, except for sorghum [4].

The consumption of oat products is increasing due to numerous health benefits associated with eating oats [1,3,6], increasing the demand and price of oat grains. It is possible to increase the total quantity of oats produced by increasing the cultivation area or by improving yield by reducing the gap between actual and potential yield [5,7]. However, in addition to the environmental requirements for the growth and production of oats, the food security needs of the growing population, the reduction of arable land and the effects of climate change also affect how many hectares can be easily converted for oat cultivation [8]. Improving cultivation practices such as improving soil fertility, optimizing fertilization management, improving tillage, and using improved varieties in oat cultivation can increase the yields and total amount of oats produced [9].

Regarding fertilization, nitrogen (N) is one of the most important nutrients in crop cultivation. It affects physiological processes and leads to morphological and quantitative changes in plant growth and development [10]. Compared to other cereals, the application of N and its effects on oat production and grain quality are less studied [11]. However, generally, an increase in yield is observed with the addition of synthetic N, but only within a certain range, usually up to 100 kg N/ha. Exceeding this range increases lodging, resulting in yield and quality losses [12,13,14].

Studies have also shown that synthetic N fertilizer (applied during plant growth) alters the stable N isotope ratio in the grains [15], i.e., the *δ*^15^N signatures differ according to the type of fertilizer. For example, synthetic fertilizers have *δ*^15^N values close to atmospheric N_2_ (0‰), while organic fertilizers have higher *δ*^15^N values [16]. For this reason, the *δ*^15^N value is the best isotopic marker for distinguishing and authenticating crop cultivation practices [16]. Although using an organic N fertilizer source does not explicitly guarantee that the crop is organically produced, synthetic fertilizers imply conventional cultivation. The *δ*^15^N values in organic crop products vary from +0.3‰ to +14.6‰, while the *δ*^15^N values in conventional crops vary from −4.0‰ to +8.7‰ [15]. The discriminatory power of *δ*^15^N has been described in several studies, and the values are influenced by the botanical plant species and the bacterial activity associated with its growth [17], the geographical origin [18] and the agricultural practices used [15,19]. Although there is limited information for *δ*^15^N values in oat grains, *δ*^13^C values have been successfully used to identify salt-tolerant genotypes of forage oats [20]. However, the use of *δ*^13^C as a marker for organic and conventional production has shown variation between studies, suggesting that *δ*^13^C values are generally independent of fertilizer source or do not vary sufficiently to distinguish the production system [21,22,23].

In addition to yield, the quality of oats, especially their protein content, is improved by the proper application of N [24,25]. Unfortunately, there is limited information on the effects of fertilization practices on elemental composition. Elements are important components of human nutrition and are responsible for maintaining health and organ function [26]. As mentioned earlier, oat grains mostly originate from extensive cultivation in marginal areas [4] and contain higher amounts of essential elements for human nutrition compared to other cereals [27,28]. The effects of the intensification of oat cultivation by increasing the N rate applied on the composition and concentration of various elements in oat grains remain unknown. Studies generally show little difference in the elemental composition of grains when organic and conventional production are compared in the same environment, on the same soil, using the same genotype [29,30,31]. The small decrease in elemental concentrations in grains of modern high–yielding varieties is due to a “dilution effect” of increased carbohydrate synthesis, which mainly affects their copper content [32]. In contrast, fertilization with synthetic N, especially ammonium-containing fertilizers, promotes the accumulation of iron (Fe) and zinc (Zn) in wheat grains [33,34].

The present study, therefore, aimed to quantify the multiple effects of synthetic N rate (0, 55, 110 and 165 kg/ha) and agronomic management practices (i.e., control without organic amendment, incorporation of manure every third year, and incorporation of crop residues/cover crops) applied in the long-term field experiment on the agronomic traits (grain yield and yield components), multi-elemental composition and isotopic signature (*δ*^15^N, *δ*^13^C) of spring oats.

## 2. Results

### 2.1. Effect of N Rate and Agronomic Management Practice on the Agronomic Performance of Oats

The mixed model ANOVA test results and the performance data for the model for agronomic performance of oats are presented in Table 1. There were significant differences between treatments with respect to grain yield (*p* ≤ 0.001), straw yield (*p* ≤ 0.001), plant height (*p* ≤ 0.001), TKW (thousand kernel weight; *p* ≤ 0.001 and *p* = 0.036, respectively) and plant lodging (*p* ≤ 0.001) in both trials (i.e., Trial 1 and Trial 2). However, the HI (harvest index) did not differ regarding the different treatments used in either trial (*p* = 0.482 and *p* = 0.245, respectively).

**Figure 1:**
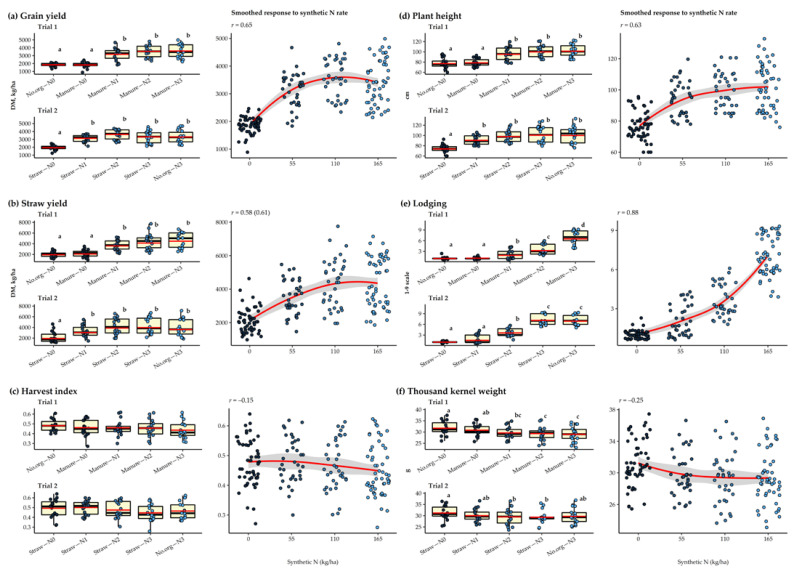
The response of oat agronomic performance traits (**a**–**f**) to different N rates and agronomic management practices applied in a long-term field experiment during 2015–2020. The red crossbar in the boxplot indicates the mean, while the dots represent individual observations. Means marked with different letters represent significant differences within each trial according to Tukey’s HSD test (*α* = 0.05). The smoothed conditional means method (Loes method) represents the overall observed response of the selected variables to the synthetic N rate and the correlation coefficient. When the data was transformed in both trials, the “*r*” value in brackets indicates the correlation coefficient for the transformed data.

Fertilization with synthetic N increased grain yield, straw yield, plant height, and plant lodging and decreased TKW regardless of the agronomic management practices of the long-term field experiment (Figure 1). Grain yields increased up to 110 kg/ha N added (N2 rate), showing a moderately strong positive correlation (*r* = 0.65). Statistical differences were observed only between those treatments where synthetic N was and was not added. The mean grain yield ranged from 1826 kg/ha (no.org–N0) to 3593 kg/ha (manure–N3) in Trial 1 and from 1936 kg/ha (straw–N0) to 3623 kg/ha (straw–N2) in Trial 2. Straw yield increased up to the highest N rate (165 kg/ha N; N3 rate), also showing a moderately strong correlation (*r* = 0.61). Again, only the treatments with and without the addition of synthetic N were significantly different. In Trial 1, the mean straw yield ranged from 1933 kg/ha (no.org–N0) to 4483 kg/ha (manure–N3), while in Trial 2 from 1921 kg/ha (straw–N0) to 3972 kg/ha (straw–N3). Comparing management practices showed a small residual effect of manure applied two years prior to the cultivation of oats in a straw yield of 115.9% and 109.9% higher than for control without organic amendment or for straw incorporation, respectively. Plant height increased up to the N3 rate and correlated moderately strongly with N rate (*r* = 0.63). Again, statistical differences were only found between treatments with and without the addition of synthetic N. The smallest plants were observed for no.org–N0 (77 cm) and the largest for manure–N3 (101.2 cm) in Trial 1 and for straw–N0 (73.8 cm) and straw–N3 (100.6 cm) in Trial 2. There was also a strong positive correlation (*r* = 0.88) between plant lodging and N rate. In Trial 1, estimates for lodging ranged from 1.0 (no lodging; manure–N0, no.org–N0) to 6.5 (manure–N3) and in Trial 2 from 1.0 (straw–N0) to 7.1 (straw–N3). 

The results also show how TKW decreases with an increasing application rate of synthetic N; however, only a weak correlation with the N rate was observed (*r* = –0.25). In Trial 1, TKW ranged from 29.0 g (manure–N3) to 31.7 g (no.org–N0). Manure–N2 and manure–N3 had a significantly lower TKW than no.org–N0 and manure–N0. In Trial 2, TKW ranged from 29.3 g (straw–N3) to 31.2 g (straw–N0) and differed significantly only between the two treatments. No significant differences were found between treatments for HI. The mean values of HI ranged from 0.44 (manure–N3) to 0.48 (no.org–N0) for Trial 1 and from 0.45 (straw–N3) to 0.50 (straw–N1) for Trial 2.

### 2.2. Effect of N Rate and Agronomic Management Practice on the Multi-Elemental Composition in Oat Gains

The multi-elemental composition was obtained for oat grains from two consecutive cultivation seasons (2016–2017). Twelve different elements were determined in 60 samples of oat grains. The samples were classified into two groups: macroelements (>1 g/kg DW) P, S, Cl, K, Si and Ca, and microelements (>0.1 mg/kg DW) Fe, Zn, Ti, Rb, Br and Sr. The mixed model ANOVA test results and the performance data for the model for macro-elements in oat grains are presented in Table 2 and for microelements in Table 3. In both trials, differences between treatments were observed for Ca (*p* = 0.045 and *p* = 0.002, respectively) and Zn (*p* = 0.011 and *p* = 0.010, respectively). Differences in the levels of Si were observed only in Trial 2 (*p* = 0.002), and Fe (*p* = 0.044) and Ti (*p* = 0.010) only in Trial 1 and Trial 2, respectively. Significant differences between sampling years were observed for the macroelements Si (*p* = 0.002, Trial 2) and Ca (*p* < 0.001, Trial 2) and microelements Fe (*p* = 0.027 and *p* < 0.001, Trial 1 and Trial 2, respectively), Rb (*p* = 0.042 and *p* < 0.011, Trial 1 and Trial 2, respectively) and Sr (*p* = 0.009 and *p* < 0.008, Trial 1 and Trial 2, respectively).

**Figure 2:**
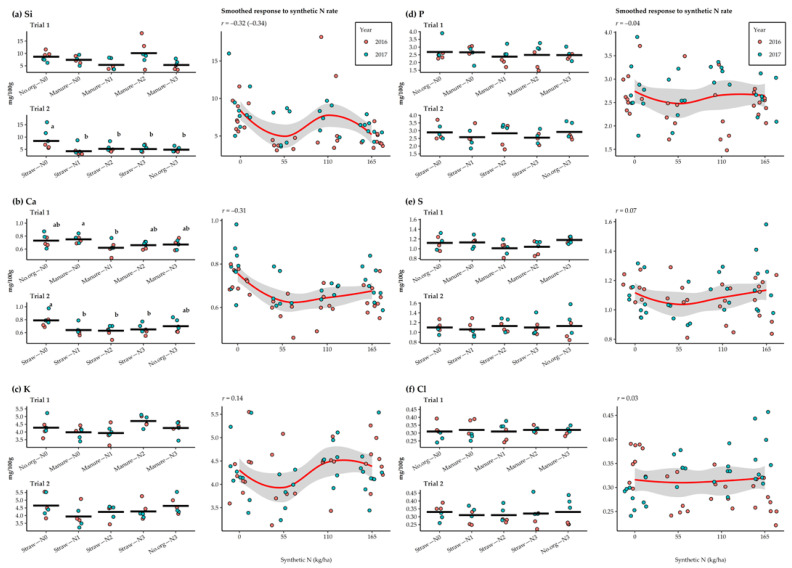
Response of macroelements (**a**–**f**) in oat grains to N rates and agronomic management practices applied in a long-term field experiment in 2016–2017. The crossbar indicates the mean value, while the dots represent individual observations. Means marked with different letters represent significant differences according to Tukey’s HSD test (*α* = 0.05) within each trial. The smoothed conditional means method (Loes method) represents the overall observed response of the selected variables to the synthetic N rate and the correlation coefficient. When the data was transformed in both trials, the “*r*” value in brackets indicates the correlation coefficient for the transformed data.

During 2016–2017 (Figure 2, the Ca content decreased with the addition of synthetic N; however, only a weak negative correlation with the N rate was observed (*r* = −0.31). In Trial 1, the Ca content was the lowest in manure–N1 (0.62 mg/100 g) and the highest in manure–N0 (0.75 mg/100 g), with significant differences between the two treatments. In Trial 1, the mean contents in 2016 and 2017 were 0.67 mg/100 g and 0.71 mg/100 g, respectively. In Trial 2, the Ca content ranged from 0.63 mg/100 g (straw–N2) to 0.79 mg/100 g (straw–N0). Here, oat grains grown in straw–N0 had a higher Ca content than in straw–N1, straw–N2 and straw–N3. In Trial 2, higher Ca content of 0.73 mg/100 g was observed in the grains in 2017 compared to 0.63 mg/100 g in 2016. It is noteworthy that comparing agronomic management practices showed a higher Ca content in the grains with incorporation of straw residues than controls without organic amendment or manure incorporation every third year. The Si content was higher in the treatments where synthetic N was not added but with only a weak correlation with N rate (*r* = −0.34) and higher variability in the data. In Trial 1, the lowest Si contents were recorded in manure–N3 (5.34 mg/100 g) and the highest in manure–N2 (10.12 mg/100 g) and in Trial 2 in straw–N1 (4.27 mg/100 g) and straw–N0 (8.42 mg/100 g). Lower Si content was observed in 2016 (5.59 mg/100 g and 4.63 mg/100 g) than in 2017 (7.31 mg/100 g and 6.39 mg/100 g) in both trials, respectively. Comparing agronomic management practices showed a 115.5% decrease in the Si where manure was added two years before for N0 and a 109.2% increase for N3 compared to controls without organic amendment.

Macroelements K, P, S, and Cl did not differ among synthetic N rate and agronomic management practices or sampling years. In addition, no correlations were found between the different N rates. The K content ranged from 3.93 mg/100 g (manure–N1) to 4.71 mg/100 g (manure–N2) and from 3.94 mg/100 g (straw–N1) to 4.65 mg/100 g (straw–N0) for Trial 1 and Trial 2, respectively. The mean K content for 2016 and 2017 ranged from 4.29 mg/100 g to 4.40 mg/100 g, showing only small variation between years and trials. The P content ranged from 2.38 mg/100 g (manure–N1) to 2.68 mg/100 g (no.org–N0) and from 2.55 mg/100 g (straw–N3) to 2.92 mg/100 g (no.org–N3), respectively. Only small differences in P were observed between sampling years, with content in 2016 of 2.32 mg/100 g (Trial 1) and 2.63 mg/100 g (Trial 2), and in 2017 of 2.75 mg/100 g (Trial 1) and 2.87 mg/100 g (Trial 2). Manure–N1 (1.01 mg/100 g) in Trial 1 and straw–N1 (1.06 mg/100 g) in Trial 2 produced the lowest S contents in the grains and the highest for manure–N3 (1.18 mg/100 g) and 1.13 mg/100 g (no.org–N3, straw–N3). The mean S content for 2016 and 2017 ranged from 1.08 mg/100 g to 1.12 mg/100 g, showing only small variation between years and trials. The Cl content varied between 0.31 mg/100 g and 0.33 mg/100 g in both trials, while for years 2016 and 2017 mean contents of 0.32 mg/100 g and 0.31 mg/100 g and 0.29 mg/100 g and 0.37 mg/100 g were detected in Trial 1 and Trial 2, respectively.

**Figure 3:**
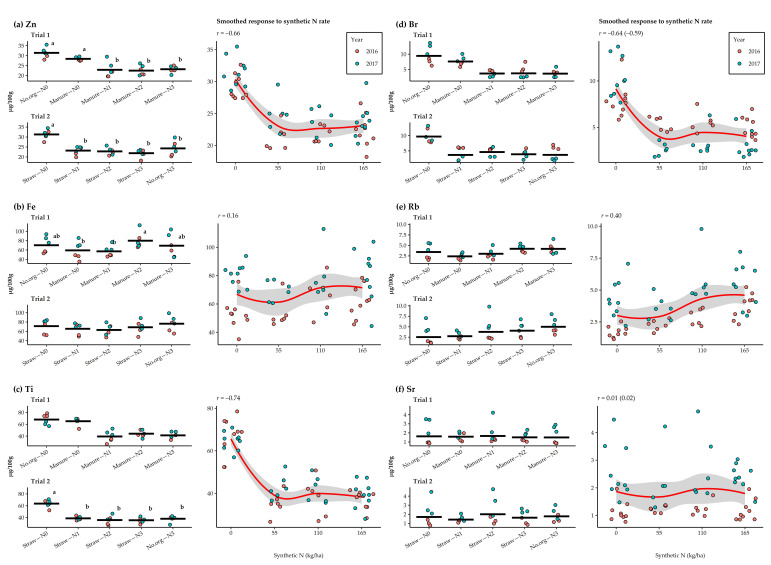
Response of microelements (**a**–**f**) in oat grains to N rates and agronomic management practices applied in a long-term field experiment in 2016–2017. The crossbar indicates the mean value, while the dots represent individual observations. Means marked with different letters represent significant differences according to Tukey’s HSD test (*α* = 0.05) within each trial. The smoothed conditional means method (Loes method) represents the overall observed response of the selected variables to the synthetic N rate and the correlation coefficient. When the data was transformed in both trials, the “*r*” value in brackets indicates the correlation coefficient for the transformed data.

The response of microelements content in oat grains to synthetic N rate and different agronomic management practices of the long-term field experiment in 2016 and 2017 is shown in Figure 3. Zn content generally decreased with the application of synthetic N, regardless of the agronomic management practices of the long-term field experiment. Significant differences were only observed between treatments with and without the addition of synthetic N, where the application of synthetic N decreased the Zn content by 124.4% on average. A strong negative correlation was observed between N rate and Zn content in grains (*r* = –0.66). In Trial 1, the Zn contents ranged from 22.5 μg/100 g (manure–N2) to 31.4 μg/100 g (no.org–N0) and in Trial 2 from 21.7 μg/100 g (straw–N3) to 31.3 μg/100 g (straw–N0). In both trials, the content of Zn was lower in 2016 (24.5 μg/100 g and 23.4 μg/100 g) than in 2017 (26.8 μg/100 g and 25.7 μg/100 g), respectively, however significantly differing only in Trial 2. It is noteworthy that comparing management practices showed 107.0% and 105.1% higher Zn content in grains from control without organic amendment compared to the practice of manure incorporation every third year, respectively. A very weak positive correlation was observed for Fe with synthetic N (*r* = 0.16). The Fe content in Trial 1 was highest for manure–N2 (79.9 μg/100 g) and lowest for manure–N1 (57.1 μg/100 g), with significant differences between the two treatments. In Trial 2, the Fe content in the oat grains ranged from 63.0 μg/100 g (straw–N2) to 76.4 μg/100 g (no.org–N3). Grains in both trials had significantly higher Fe content in 2017 (78.3 μg/100 g and 77.0 μg/100 g) compared to 2016 (55.9 μg/100 g and 61.0 μg/100 g), respectively. Comparing management practices showed that the higher Fe contents were found in grains cultivated without organic amendment (in the long-term experiment this is applied with removal of aboveground biomass and no manure incorporation). The Ti content also decreased with the addition of synthetic N, regardless of the long-term experiment agronomic management practices applied, and differed only between treatments with and without synthetic N. A strong negative relationship was observed between N rate and Ti content in the grains (*r* = –0.74). In Trial 1, the Ti content ranged from 41.3 μg/100 g (manure–N3) to 68.0 μg/100 g (no.org–N0). In Trial 2, the Ti content ranged from 34.1 μg/100 g (straw–N3) to 63.3 μg/100 g (straw–N0). Lower Ti content in Trial 2 was observed in both years, ranging from 39.5 μg/100 g in 2016 to 42.3 μg/100 g in 2017, and in Trial 1 ranging from 50.6 μg/100 g in 2016 to 52.5 μg/100 g in 2017.

The Br content showed no significant difference among years and N rates and management practices, while the microelements Rb and Sr were significantly affected by the year. In general, Br content decreased with the application of synthetic N, regardless of the N rate and agronomic management practices applied in the long-term experiment. A moderately strong negative correlation was observed between synthetic N rate and Br content (*r* = –0.59). The decrease in mean Br content when synthetic N was applied was 154.9%, and Br content was lowest where manure was incorporated two years before cultivation of oat. Mean content of Br in year 2016 and 2017 were 5.5 μg/100 g and 4.8 μg/100 g in Trial 1 and 6.2 μg/100 g and 3.6 μg/100 g in Trial 2. The mean Rb content was 184.1% higher in 2017, being 4.2 μg/100 g and 5.2 μg/100 g compared to 2016 2.7 μg/100 g and 2.4 μg/100 g in Trial 1 and Trial 2, respectively. The mean Rb content was 184.1% higher in 2017. Rb content generally increased with the amount of N applied, but there was only a weak positive relationship with the N rate (*r* = 0.40). Comparing agronomic management practices showed that incorporation of manure two years before oat cultivation or incorporation of straw residues decreased the Rb content in grains by 123.3% and 122.2%, respectively, compared to control without organic amendment. For Sr, the average contents in the grains were 202% higher in 2017, with values in both trials ranging from 2.3 μg/100 g a to 2.4 μg/100 g, while 2016 ranging from 1.1 μg/100 g to 1.2 μg/100 g, respectively. However, correlation analysis showed no relationship between N rate and Sr content (*r* = 0.02).

### 2.3. Effect of N Rate and Agronomic Management Practice on the Stable Isotope Ratio of N and C in Oat Grains

The stable isotope composition (*δ*^15^N, *δ*^13^C) of oat grains was analyzed in the 2016 and 2017 growing seasons. The results of the mixed model ANOVA test and the performance of the model for *δ*^15^N and *δ*^13^C values in oat grains are shown in Table 4. Here, significant differences were found between treatments for *δ*^15^N (*p* = 0.040 and *p* = 0.039, respectively) and *δ*^13^C values (*p* = 0.031 and *p* = 0.006, respectively) for both Trial 1 and Trial 2. However, the cultivation year did affect the *δ*^13^C values in both trials (*p* = 0.004, and *p* < 0.001, respectively).

**Figure 4:**
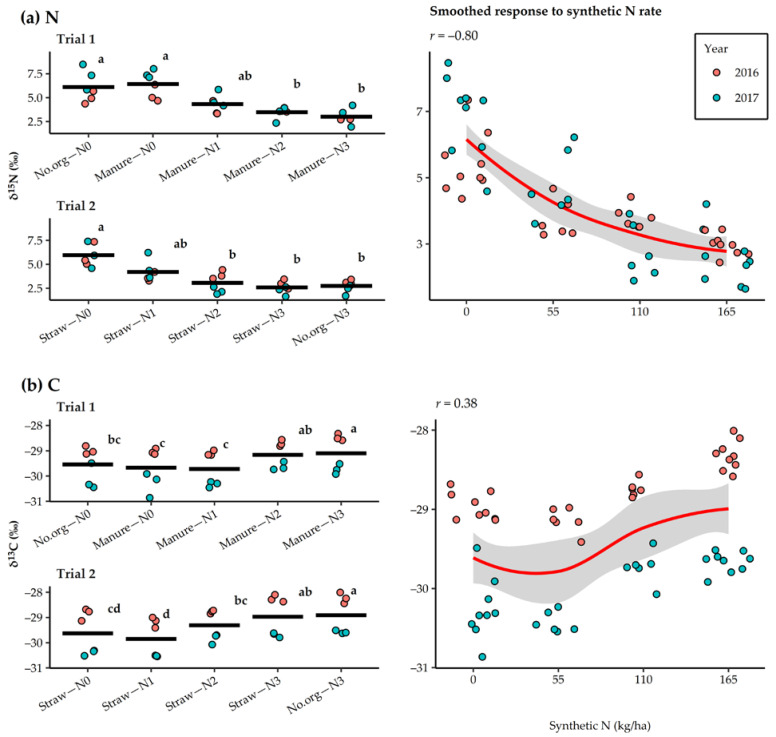
Response of *δ*^15^N and *δ*^13^C values (**a**,**b**) in oat grains to N rates and agronomic management practices applied in a long-term field experiment in 2016–2017. The crossbar indicates the mean value, while the dots represent individual observations. Means marked with different letters represent significant differences according to Tukey’s HSD test (*α* = 0.05) within each trial. The smoothed conditional means method (Loes method) shows the overall observed response of the selected variables to the synthetic N rate and the correlation coefficient.

Figure 4 shows the response of *δ*^15^N and *δ*^13^C values in oat grains to N rate and agronomic management practices of the long-term experiment in 2016 and 2017. A strong negative correlation was observed between synthetic N rate and *δ*^15^N values (*r* = –0.80). In general, the application of synthetic N decreased *δ*^15^N values up to the highest rate, regardless of the management practice. However, significant differences in grain *δ*^15^N values were found only between treatments in which synthetic N was not added and those in which 110 or 165 kg N/ha was applied. In Trial 1, the *δ*^15^N values ranged from 3.0‰ (manure–N3) to 6.4‰ (manure–N0) and in Trial 2 from 2.6‰ (straw–N3) to 6.0‰ (straw–N0). In 2016 the *δ*^15^N values were 4.1‰ and 3.9‰ and in 2017 5.2‰ and 3.5‰ in Trial 1 and Trial 2, respectively. Comparing management practices showed small residual effect of applying manure two years before cultivation of oats by increasing the *δ*^15^N values in the oat grains by 107.6% and 110.2% compared to control without organic amendment and straw incorporation, respectively. The *δ*^13^C values in the grains generally increased with the synthetic N rate, up to the highest rate, regardless of the management practice. However, correlation analysis revealed only a weak positive relationship between synthetic N rate and *δ*^13^C values (*r* = 0.38). In Trial 1, *δ*^13^C values ranged from –29.7‰ (manure–N1) to –29.1‰ (manure–N3). Values for manure–N3 were significantly higher than those for manure–N1, no.org–N0, and manure–N0. In Trial 2, the *δ*^13^C values ranged from –29.9‰ (straw–N1) to –28.9‰ (no.org–N3). The *δ*^13^C value was significantly higher in no.org–N3 than in straw–N2, straw–N1 and straw–N0. In both trials significantly higher *δ*^13^C values were observed in 2016 (−28.9‰ and −28.7‰) than in 2017 (−30.0‰), respectively.

### 2.4. Multivariate Analysis

**Figure 5:**
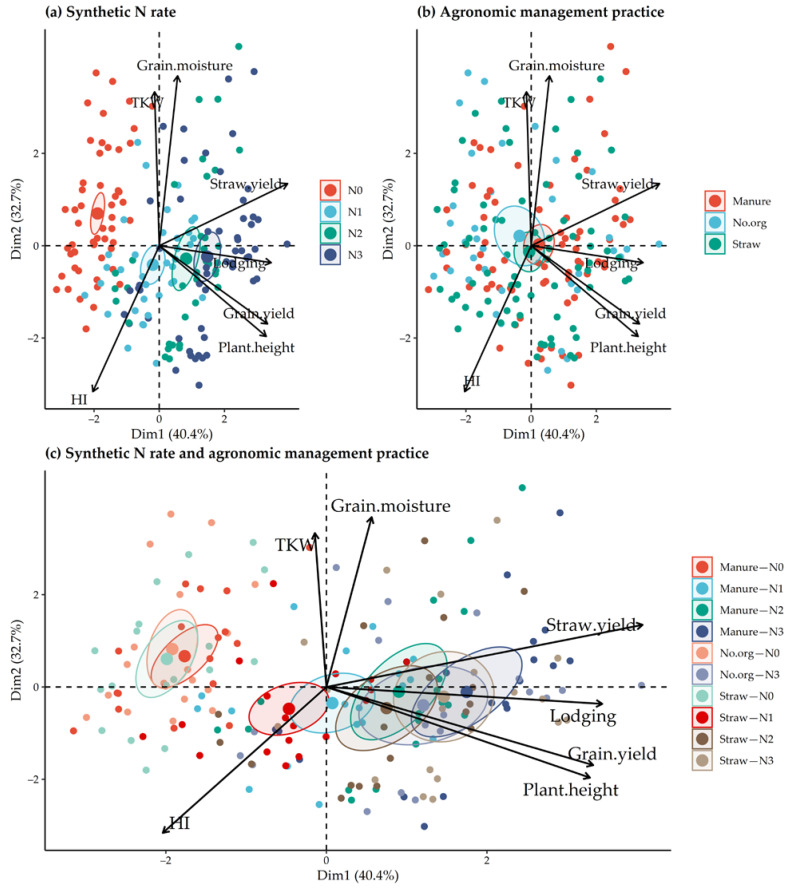
PCA analysis score plots (**a**–**c**) of oat agronomic performance traits (grain and straw yield, grain moisture, plant height, plant lodging, TKW and HI) during 2015–2020 as a function of applied N rate and agronomic management practices of the long-term field experiment for the first and second principal components. The ellipses represent 95% confidence intervals.

**Figure 6:**
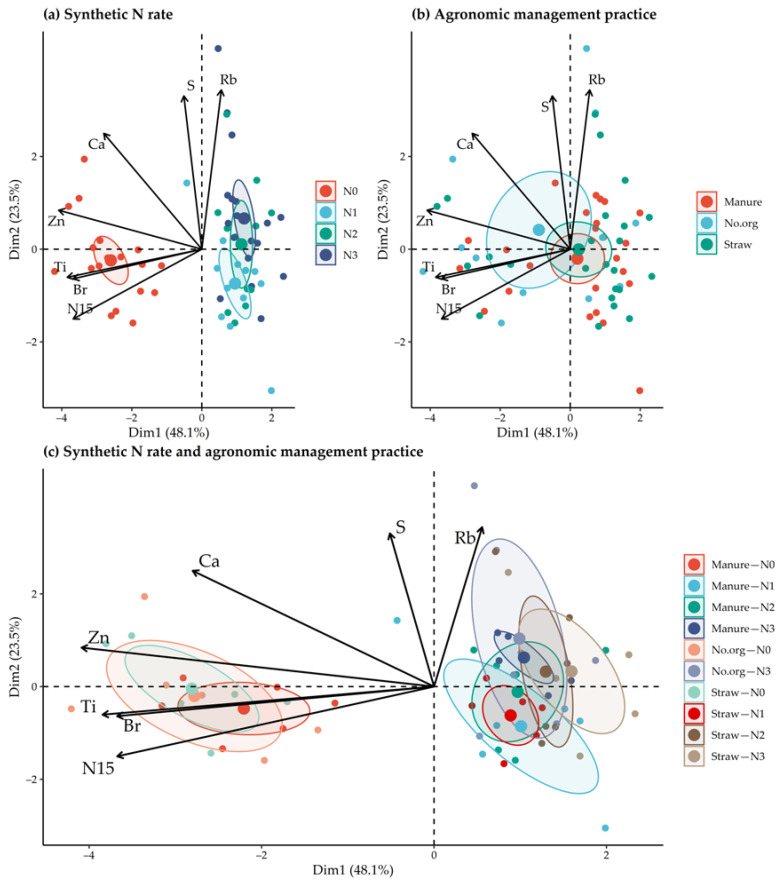
PCA analysis score plots (**a**–**c**) of macroelements (Ca, S), microelements (Ti, Zn, Rb, Br) and *δ*^15^N values in oat grains from 2016–2017 as a function of applied N rate and agronomic management practices of the long-term field experiment for the first and second principal components. The ellipses represent the mean and 95% confidence interval.

For the data on agronomic traits (Figure 5) and on the elemental and stable isotopic composition of grains (Figure 6), separate PCA was performed according to (a) synthetic N rate, (b) agronomic management practices of the long-term experiment, and (c) combinations of synthetic N rate and agronomic management practice. For agronomic traits, the total contribution to the first two components of variation was 73.1%. The first principal component (PC1) contributed 40.4% to the total variation and correlated with grain yield, straw yield, lodging and plant height. The second principal component (PC2) contributed 32.7% to the variation and revealed a correlation with TKW, grain moisture at harvest and HI. Oats grown without synthetic N differed significantly from those grown with synthetic N in terms of agronomic traits (Figure 5), while it was impossible to distinguish between different agronomic management practices of the long-term field experiment. The combination of organic amendment and synthetic N fertilization also shows the strong influence of the synthetic N on the selected agronomic traits. The most influential agronomic parameters for differentiating between N rate and agronomic management practice in oats were grain yield, straw yield, lodging, and plant height.

PCA analysis using all elemental and *δ*^15^N and *δ*^13^C data showed that PC1 contributed 29.3% to the variations and correlated with Zn, Ca, Ti and Br while PC2 accounted for 20.0% to the variation and correlated with Rb, *δ*^15^N, Ti, Br, and S. Dimensionality reduction, by removing the variables with the least variance, increased the overall contribution to the first two components of variation to 71.5% (Figure 6). Oat grain composition was differentiated by synthetic N use, but only between with and without the synthetic N used for plant growth. Differentiation by the management practices of the long-term experiment was not possible. A strong effect of synthetic N use on elemental and isotopic composition was also observed for combinations of agronomic management practice synthetic N fertilizers. The higher contents of Zn, Ti, Br and *δ*^15^N distinguished oat grains from the treatments without synthetic N from those in which synthetic N was applied during growth.

## 3. Discussion

Improvements in plant genetics and the use of synthetic N fertilizers have significantly increased cereal production over the past 50 years [35,36]. However, oats have received less attention from breeders, researchers, and even agronomists, making them less competitive than other cereals in high–yielding environments [37]. In the present study, the addition of synthetic N to oats resulted in morphological and physiological changes in plant growth regardless of the agronomic management practices applied in the long-term field experiment. In contrast, agronomic management practices did not affect oat performance in the long-term field experiment. In the oat cultivar ‘Noni’, limited intensification of N fertility was possible as the grain yield increased with the addition of synthetic N up to a value of 110 kg/ha. Above this, a further increase in N rate resulted in significant plant lodging and associated loss of harvestable grain yield. According to other studies, the optimum N rate for oats ranges from 30 to 120 kg/ha depending on the environment, cultivar used, crop rotation and tillage applied [12,13,14,38,39,40,41,42]. There has been a steady increase in the optimum N rate for oats, with recent studies generally reporting optimum N rates for oats in excess of 100 kg/ha [12,38,42]. The results of the present study are consistent with these findings. However, direct comparisons of studies are difficult due to environmental and methodological differences. For example, da Silva et al. [42] observed similar grain yields in oats as in the present study with the addition of a 120 kg/ha of synthetic N in soybean/oat rotation, while yields in maize/oat rotation (a better approximation of the rotation in the long-term experiment here) were ~45% lower. The ability of oat cultivars to use supplemental N to improve grain yield varies greatly and should be considered when determining optimal N rates. In this example, the ability of 120 cultivars to use added N to improve grain yield was highly variable. When 60–90 kg N/ha was used, yields ranged from 1790 to 5990 kg/ha [43]. It is noteworthy that the highest yielding cultivars were the improved cultivars from the European breeding programs. May et al. [44] also observed differences among cereal cultivars in yield responses to N rate. They observed a linear increase in grain yield of up to 120 kg/ha of synthetic N in one cultivar, and a curvilinear increase peaking at about 80 kg/ha of synthetic N in another. Plant reproductive performance can also be determined from the HI, defined as the ratio of grains to total shoot dry matter and indicates efficiency in converting biomass into grain yield [45]. The HI can be influenced by interactions between genotype (G), environment (E) and crop management (M) [46]. In the present study, oats HI was not influenced by the N rates and management practices of the long-term field experiment, while the random effect of year was responsible for most of the observed variation. Similar results were obtained in spring wheat, where N fertilization or the cultivar used did not affect HI, while HI was more environmentally dependent [45].

In the present study, an increase in plant lodging was the most negative effect of increasing the synthetic N rate. The susceptibility of oat plants to lodging is considered the main obstacle to improving grain yield by adding N [14]. In the present study, the highest amount of N applied resulted in more than 90% of the plants being fully lodged in most growing seasons, resulting in a significant loss of harvestable grain. It is noteworthy that a special micro-plot harvester was used so that a large proportion of lodged plants could be harvested, whereas the authors suspect that this would not be possible to the same extent with standard agricultural harvesting machinery on all fields. Recent studies have reached similar conclusions to those of the present study, generally reporting significant increases in lodging at N rates above 100 kg/ha [38]. Differences between cultivars have also been reported, with tolerant cultivars tolerating 20–30 kg higher synthetic N applications [25,44]. Newly released oat cultivars are thought to be more resistant to lodging due to the introduction of dwarfing genes [47,48] although cultivars with dwarfing gene *Dw6* have been available for more than 30 years [5,49]. Eight dwarfing genes have been identified and studied in oats, however only *Dw6, Dw7* and *Dw8* have been used for plant improvement, with *Dw6* as the most widely used among them [49]. In contrast to previously reported results [25], in the present study, genetic gains in shortening oat plants were not lost with the increase in synthetic N rate, as plants showed no relevant differences in height between 55 and 165 kg/ha of N addition.

In addition to genotype and fertilization, site-specific factors also affect the risk of lodging and associated N management in oats. For example, warmer growing conditions resulted in taller stems with thinner cell walls and consequently higher susceptibility to lodging, which affected N management at this site [38]. The same can be confirmed in the present study, although the year was considered a random factor. Anchorage failure is considered the main cause of lodging in oats [14]. However, this was not the case in the present study, where plant lodging increased significantly due to decreased stem stiffness associated with increasing synthetic N application. Increasing oat biomass likely exceeded internode strength at the highest N rate, suggesting that internode strength was either reduced or too low to maintain plant biomass. There are several management strategies to reduce the susceptibility of oats to this type of lodging, however, with results varying according to the intensity of N application and environment [14,50].

The application of synthetic N reduced Ca, Zn, Ti, Si, and Br contents and increased Fe and Rb content in the oat grains. Differences were observed only between treatments with and without the addition of synthetic N while increasing the N rate from 55 kg/ha to 165 kg/ha did not reduce element contents. On the contrary, macroelements K, P, S and Cl and microelement Sr were not affected by N fertilization. Comparing agronomic management practices of the long-term field experiment showed little difference in the elemental composition of the grains. Ryan et al. [31] reported lower Zn and Cu contents and higher Mn and P contents in conventionally grown wheat grains compared to organically grown. It is noteworthy that there are no true organic treatments present in this long-term field experiment (only treatments without synthetic N), while comparing results of conventional cultivation a similar decrease in Zn content due to synthetic N was observed in oat grains. However, results of the present study contrast with recent studies on wheat grains where synthetic N fertilizer increased the accumulation of Fe, Zn, and Cu [33,34]. As reported, the improvement in Fe and Zn content of wheat grains by synthetic N application was related to the increased sink strength of the grains for Fe and Zn and positively correlated with the amount of protein in the grains [33]. Although these studies suggest that appropriate N management improves the content of certain microelements in wheat grains, contrary results for spring oats from the present study suggest that this may vary and depend on environmental conditions, management practices, or perhaps even the species grown. In addition, this study shows that although total Zn uptake and accumulation in grains per plot improved with the increase in N rate, grain Zn content per 100 g DM decreased. This suggests a dilution effect of Zn with the intensification of oat cultivation, where improved dry matter production is not accompanied by a proportional increase in grain Zn content at higher N rates. In general, the elemental composition of grains is related to the concentrations and availability of certain nutrients in the soil, the ability of the genotype to utilize and translocate them, environmental conditions for plant growth, and management practices. These factors can significantly affect the elemental composition of grains within and between closely related species, leading to varying results in published studies. The *δ*^13^C value in oat grains (discussed further below) shows that growing season 2017 was more favorable for spring oats at Jablje, as also shown by slightly higher grain yield in that year. Possibly, higher content of some of the analyzed elements in grains from the same year also reflected this finding. However, as oats were grown in different main plots of the long-term field experiment, the difference observed between years for the contents of some of the elements probably reflects differences for the elements in soil. The present study analyzed the elemental composition of oat grains in the 23rd and 25th year of a long-term field experiment. Since the elemental composition and the total amount of elements accumulated in oat grains reflect their availability in the soil, no evidence of soil nutrient depletion at elevated N rates compared to the control plots without the application of synthetic N during this period were found. These results are consistent with the review by Marles [32]. 

This study was started in the 22nd year of a long-term field experiment with well-established differences in soils between agronomic management practices. The drawbacks of conducting the study in the framework of an existing experiment are the specifics of the experiment that need to be maintained for consistency. In general, treatments in the long-term experiment consist of combinations of N rate and management practices (i.e., no organic amendment, manure incorporation every third year, and straw/cover crop incorporation), while the timing of the implementation of combinations differs for some crops in the rotation. For example, manure is applied only once per three growing seasons before maize, while for the second (winter wheat) and third (spring oat) seasons, only synthetic N is applied to these plots. In contrast, aboveground biomass is always removed on the plots without organic amendment and on the plots where manure is added and always ploughed into the plots with straw incorporation management practice. This results in unique combinations of different direct and residual effects of synthetic N fertilization and management practices for crops in the rotation. The majority of the differences in oat agronomic performance, elemental composition and isotope values reported in this study were caused by the use of synthetic N. In contrast, agronomic management practices of the long-term experiment in general showed only small effects on oats. Based on the management and fertilization specifics of the oats as a third crop in the rotation of the long-term experiment, the residual effect of manure N on the oats agronomic performance was only small, as also demonstrated by the grain *δ*^15^N values (discussed further below). Incorporation of straw with its wide C:N ratio also showed only small effects on the oat agronomic performance. While small effects of management practices on the agronomic performance of oats was assumed based on the mentioned management specifics of the experiment and oats as the third crop in the rotation, for the elemental composition and isotope values, differences were expected as management practices have affected soil organic matter/carbon content, and the content of elements in the soil of the long-term experiment. For the duration of the long-term experiment all plots received the same amount of P and K with fertilizers, while various macro and microelements were added to the soil with manure or cycled in their availability and amount with straw incorporation, and on the contrary were removed from the soil with removal of aboveground biomass in the management without organic amendment and manure incorporation. Soil analyses of the long-term experiment show that applying these practices has changed the content of the analyzed elements. For example, analysis in 2020 shows difference in the content of available P_2_O_5_ and K_2_O between management practices, with the highest P_2_O_5_ content with the use of manure, while a build-up of K_2_O is noted with incorporation of straw. It has been reported in other studies that significant build-up of K in soil can occur by combining straw incorporation and K fertilizers [51], and this can be confirmed here. Management practice of straw incorporation supported with K fertilization improved the content of available K_2_O in the top 25 cm of soil from 138 mg/kg at the start of the experiment to 231 mg/kg analyzed in 2020, which represents an increase to 167%. Increases of available K_2_0 content by 139% and 128% were observed for manure incorporation and no organic amendment management, respectively, indicating a surplus of K in all management practices. However, content of P_2_O_5_ decreased in the same period from 318 mg/kg at the start of the experiment to 292 mg/kg where manure is incorporated, 242 mg/kg where straw is incorporated and 227 mg/kg in controls without organic amendment. Nevertheless, these differences were not reflected in the content of K and P in oat grain in years 2016 and 2017. Management practices applied in the long-term field experiment also caused differences in soil organic matter/carbon content in the same period. For example, based on the amount of organic carbon of 51.9 t/ha at the start of the experiment, soil analysis in 2020 showed the amount in the top 25 cm of soil decreased yearly by the rate of 0.10 t/ha in plots where no organic amendment was practiced, and increased by the rate of 0.18 t/ha and 0.12 t/ha where manure or straw was incorporated, respectively. Despite this, differences in the soil organic carbon have not affected the performance of oats, possibly indicating that the differences were too small and/or the capacity of oats to tolerate these differences was high enough not to have an effect on performance.

The assumption of grain elemental composition being affected by the management practices of the long-term experiment was not supported in this study, and no relevant differences in oat grain elemental between different management systems of the long-term trial were observed. Further studies are needed to confirm or refute whether and to what extent changes in oat N management (especially intensification) might alter grain microelement content in different environments and cultivated practices. The strong negative correlation of Zn, Ti and Br with synthetic N in the present study also suggests that they have the potential to authenticate spring oat cultivation with or without synthetic N use. These results agree with those of Kelly and Bateman [52], where the combination of microelements with *δ*^15^N values improved the classification of cultivation practices, but contrast with those of Laursen et al. [53], where no single element discriminated between conventional and organic cultivation across sites, years and plant species.

Both *δ*^15^N and *δ*^13^C values in oat grains changed with synthetic N application in this study, indicating their discriminatory potential in identifying the cultivation practices used for oats. Using *δ*^15^N and *δ*^13^C values, synthetic N use, and to some extent the amount of synthetic N applied, was distinguished. In general, increasing the N rate decreased the *δ*^15^N and increased the *δ*^13^C values in the oat grains. However, statistically, only a difference between the values with and without synthetic N application above the synthetic N rate of 55 kg/ha was detected. In addition, no differences between agronomic management practices of the long-term experiment were found for any of the stable isotopes, although *δ*^15^N values in the oat grains were consistently higher where manure was used two years before cultivation of oat. To the best of the authors’ knowledge, there are no previously published results for oats on *δ*^15^N or *δ*^13^C values and their changes as a function of fertilizer application. Many studies describe the discriminatory power of δ^15^N in identifying cultivation practices of different crops [15,16], and it is now confirmed for oats in this study.

The observed reduction in *δ*^15^N values in oat grains when synthetic N is used is related to the fact that *δ*^15^N values in synthetic N fertilizers are close to 0.0‰. In contrast, organic plants supplied with animal manure and compost have higher *δ*^15^N values [19]. In the present study, when comparing agronomic management practices (i.e., the use of manure, treatments without organic amendment and incorporation of crop residues), higher *δ*^15^N values were found for manure at the same N rate in each case. Bogaard et al. [54] reported higher *δ*^15^N values in oat grains when manure was used of 2.6‰ to 8.0‰. Methodological differences in manure application, aboveground biomass management and site-specific conditions could explain the observed differences in *δ*^15^N values between the different studies. As shown, significant *δ*^15^N can occur in the first cropping season after manure application [55], while the oats in this study were grown in the third cropping season after manure application. The results of this study show that much of the *δ*^15^N from manure is already depleted by the time oats are grown. Therefore, the ability to discriminate between used and unused manure also depends on the duration of manure incorporation. In addition, differences in *δ*^15^N values between manure use and crop residue incorporation increased with increasing synthetic N inputs. Not knowing this information could affect the interpretation of data from stable isotope analysis.

Since the *δ*^13^C value can indicate plant metabolism, the potential of *δ*^13^C to discriminate between organically and conventionally grown plants has been investigated in many studies, generally finding insufficient variation to discriminate the cultivation type [18,22]. In the present study, significant discrimination of synthetic N application in oat crops was possible, but only between no/low N application and high N application. Moreover, the significant differences between years indicate a strong influence of environmental conditions on *δ*^13^C value. While the bulk plant δ^13^C value can indicate plant metabolism, site-specific environmental conditions, such as drought, solar radiation intensity, low temperature, low atmospheric pressure and ozone stress, influence *δ*^13^C values, as environmental stresses affect the stomatal conductance and carboxylation balance [15,56]. Comparison of the environmental conditions of the study shows that 2017 was warmer and with higher monthly solar radiation, while precipitation was higher in 2016, although no drought was observed in either of these years. As the *δ*^13^C value increases with increased environmental stress, the results suggest that 2017 was a more favorable growing season at Jablje for spring oats. However, based on our results, the potential to use *δ*^13^C alone as an indicator of oat cultivation practices seems limited.

## 4. Materials and Methods

### 4.1. Experimental Site

**Figure 7:**
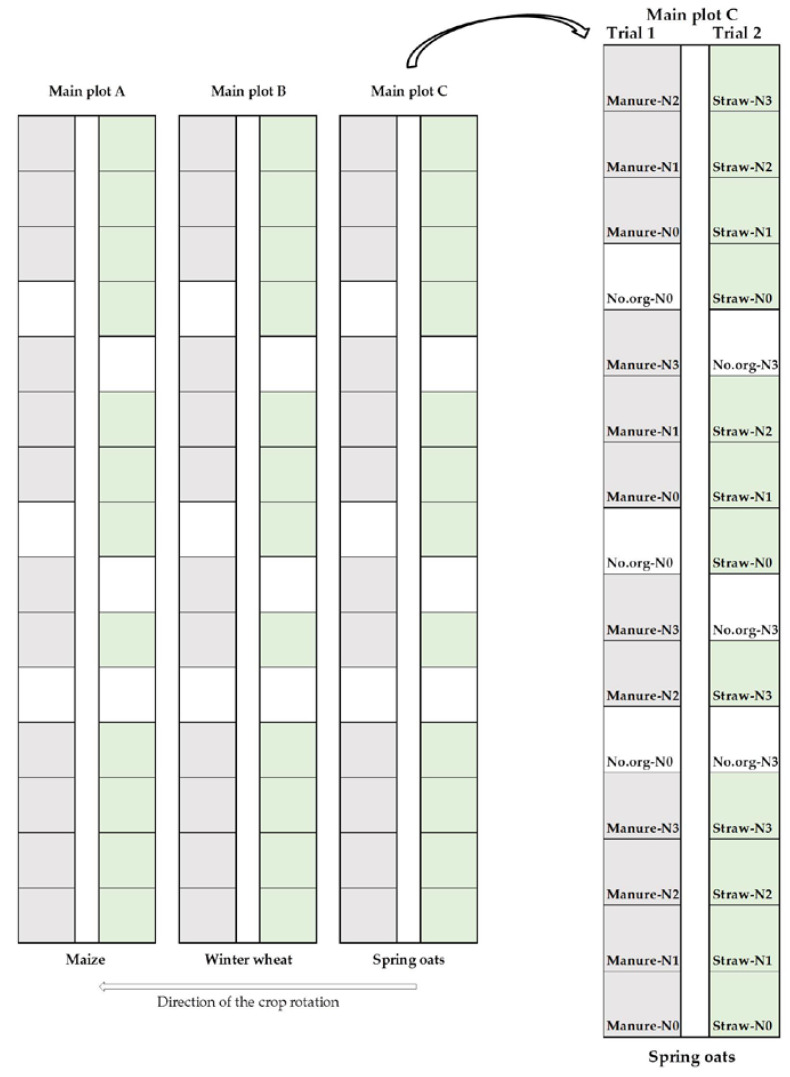
Layout and design of the long-term field experiment IOSDV at Jablje, Slovenia.

This study was conducted as part of a long-term field experiment IOSDV (International Organic Nitrogen Fertilization Experiment) at the testing station of the Agricultural Institute of Slovenia, in Jablje (46°30′17.4″ N, 15°37′34.6″ E; 320 m a.s.l., subalpine climate). The experiment was set up in 1992 with the main objective to study the long-term effects of management practices and synthetic N fertilization on soil fertility, soil organic carbon content and crop yields in rotation. A three-year rotation of maize, winter wheat and spring oats has been practiced. The IOSDV was laid out with three main plots where the crops in the rotation are grown each year. Each of the main plots was divided into two 450 m^2^ subplots (Figure 7). Each of the subplots is 90 m long and 5 m wide, with 2 m separation between subplots. The treatments for each crop are arranged within the two subplots. The basic arrangement of the IOSDV is factorial with three replications. Two main factors are agronomic management practice (control without organic amendment, incorporation of manure every third year, and incorporation of crop residues/cover crop in the rotation) and synthetic N rate. For each crop in the rotation, a total of 10 combinations of agronomic management practice and N rate were laid out in three replicates in 5 × 6 m plots. Details of each treatment, the amounts of synthetic N used, and the sequence of synthetic N application for oats are summarized in Table 5. A synthetic calcium ammonium nitrate fertilizer (27% N; 50% as ammonium-N and 50% as nitrate-N) was used for fertilizing oats. IOSDV is not fully randomized, as the incorporation of manure always occurs on the left subplot (Trial 1), and the incorporation of straw residues always occurs on the right subplot (Trial 2). In addition, control plots N0 (no.org–N0) are randomized within Trial 1, where manure is incorporated, and control plots N3 (no.org–N3) are randomized within Trial 2, where straw is incorporated.

Tillage of the experimental plots is conventional, with a 25 cm deep plough followed by seedbed preparation with cultivation. Oats are the third species in the crop rotation, after maize and winter wheat. Cattle manure (30 t/ha) is applied every third year in spring before maize cultivation. Samples of the manure used were analyzed in 2015 and 2018–2020 and were shown to contain 151–231 g/kg DM, 3.36–5.56 g/kg total N, 4.12–8.96 g/kg total K and 0.80–2.20 g/kg total P. On plots where manure is incorporated, the aboveground biomass of the crops in the rotation is always removed. Where straw is incorporated, the residue biomass of the crops in the rotation is incorporated into the soil annually. In addition, oil radish (*Raphanus sativus* var. Oleiferus) is sown as a cover crop after the oat harvest in Trial 2 and incorporated into the soil in the spring of the following year. In the treatments without organic amendment (no.org), all aboveground biomass of the plants in the crop rotation is removed annually.

The soil type is Umbrian planosol with a silt loam texture and a bulk density of 1.61 in the upper 25 cm. The analysis of the top 25 cm of soil in 2020 showed a mean pH of 5.9, and mean contents of P_2_O_5_, K_2_O, and MgO of 256, 200 and 145 mg/kg, respectively. The organic carbon content in 2020 ranged from 1.32% to 1.62%, lowest in the treatments without organic amendment and highest in a combination of straw or manure incorporation and synthetic N. Total N content in the top 25 cm averaged 0.16%. The spring oat cultivar ‘Noni’ (maintainer Agricultural Institute of Slovenia) was investigated. This cultivar was registered in 2005 and is characterized by high disease and lodging tolerance, high yield and medium late maturity with medium sized grains. The sowing density (160 kg/ha) and sowing dates (15 to 25 March) followed recommendations for the selected oat cultivar and the climatic conditions at the experimental site. Sowing was done with the plot seeder for small grains (Plotseed TC, Wintersteiger, Ried im Innkreis, Austria). The sowing date and applications of P (70 kg/ha) and K (110 kg/ha) were the same for all experimental plots. Weeds were controlled with a post-emergence herbicide (Lintur 70 WG, Syngenta, Basel, Switzerland), while an insecticide (Karate Zeon 5 SC, Syngenta, Basel, Switzerland) was used to control cereal leaf beetle (*Oulema melanopus* L.). Fungicides were not used during the study. All climatic parameters during the 2015–2020 growing season were obtained from an on-site automated weather station and are summarized in Table 6.

### 4.2. Agronomic Performance Oats and Sample Preparation

The following traits were evaluated to assess the agronomic performance of oats: grain and straw yield (kg/ha), grain and straw moisture at harvest (%), harvest index (HI), plant height (cm), thousand kernel weight (TKW; g) and lodging susceptibility. Plant height was measured on ten randomly selected individual plants in each plot before harvest. Lodging susceptibility was assessed using a 1–9 scale (1 no lodged plants, 9 all plants lodged) [57]. Oats were harvested using a plot harvester at the full maturity stage (Nursery Master, Wintersteiger, Ried im Innkreis, Austria). As individual plots in each subplot are separated with only a minimal gap, top and bottom 1.5 m of the plot borders was discarded. Each plot was then harvested by two passes of the harvester (1.5 × 3 m) for a total harvested area of 9 m^2^, where grain and straw yields were weighed. Approximately 1 kg of representative grain and straw samples were used for moisture determination. The moisture of harvested grain (%) was determined by heating at 103 °C for 4 h, and moisture of harvested straw (%) was determined by heating at 60 °C for 24 h. Grain and straw yields were calculated and expressed in kg/ha dry matter (DM). The HI was calculated as the ratio of DM grain yield to total DM of aboveground biomass. For elemental composition, stable isotopes and TKW, the air-dried (at 40 °C for 48 h) oat grains were used. All these samples were stored at 4 °C until analysis. The laboratory seed counter and an electronic laboratory-scale weight reading to 0.05 g accuracy were used to determine TKW. The oat grains to determine elements and stable isotopes were homogenized and pulverized directly before analysis using a laboratory ball mill (Retsch MM 400, Retsch GmbH, Haan, Germany) at a frequency of 30 Hz for 60 s. Here, the oat grain samples from the two consecutive years: 2016 and 2017, were analyzed.

### 4.3. Multi-Elemental Analysis Using EDXRF

In a single measurement, twelve elements (Si, P, S, Cl, K, Ca, Ti, Fe, Zn, Br, Rb, Sr) were identified using non-destructive energy dispersive X-ray fluorescence (EDXRF) spectrometry. A total of 60 homogenized oat grain samples were analyzed, 30 from 2016 and 30 from 2017. Powdered sample pellets (0.5 to 1.0 g) were prepared using a pellet die and a hydraulic press. The pellets were analyzed using an EDXRF spectrometer composed of a Si (Li) detector (Canberra, Australia), a spectroscopy amplifier (M2024, Canberra, Australia), an analogue to digital converter (M8075, Canberra, Australia), and a PC–based multichannel analyzer (S-100, Canberra, Australia). The disc radioisotope excitation sources of Fe-55 (25 mCi) and Cd-109 (20 mCi) from Eckert & Ziegler (Berlin, Germany) were used as primary excitation sources. The spectrometer was equipped with a vacuum chamber. The energy resolution of the spectrometer was 175 eV at 5.9 keV. The analysis of the X-ray spectra was performed using the AXIL (IAEA, Vienna, Austria) spectral analysis program [58]. The overall uncertainty of the spectral measurement and analysis was less than 1% in most cases. Quantification was performed using the in-house developed QAES (quantitative analysis of environmental samples) software (Jožef Štefan Institute, Ljubljana, Slovenia). The estimated uncertainty of the analysis was 5% to 10%. The quantified twelve elements were expressed as macro- (g/kg) or microelements (mg/kg).

### 4.4. Stable Isotope Ratios Analysis

Homogenized oat samples for simultaneous ^13^C/^12^C and ^15^N/^14^N isotope ratio analysis were weighed (10 mg), folded in a tin capsule (Sercon, Crewe, UK), closed with tweezers and introduced into the autosampler. The analysis was performed using an elemental analyzer coupled to an isotope ratio mass spectrometer (EA/IRMS) IsoPrime100–Vario PYRO Cube (OH/CNS Pyrolyser/Elemental Analyser; IsoPrime, Cheadle Hulme, UK). The stable isotope compositions are depicted according to the IUPAC guidelines [59,60] and expressed using the conventional *δ*-notation (‰): *δ* (‰) = [(R_sample_/R_standard_)–1] × 1000, where R is the ratio between the heavier and the lighter isotope (^13^C/^12^C, ^15^N/^14^N) in the sample and standard, respectively. Values are reported relative to the following international standards: for carbon, the Vienna Pee Dee Belemnite (VPDB) and atmospheric N_2_ (AIR) for nitrogen. The *δ*-values of the standards are defined as 0‰. To monitor precision and accuracy of measurements, the following reference materials were used: B2155 Protein Sercon *δ*^13^C = −26.98 ± 0.13‰, *δ*^15^N = 5.94 ± 0.08‰ and Casein Protein CRP *δ*^13^C = −20.34 ± 0.09‰, *δ*^15^N = 5.62 ± 0.19‰, IAEA-N-1 *δ*^15^N = 0.4 ± 0.2‰ and IAEA-N-2 *δ*^15^N = 20.3 ± 0.2 ‰ and USGS43 *δ*^13^C = −21.28 ± 0.10‰, *δ*^15^N = 8.44 ± 0.10‰. Each sample was analyzed in triplicate, and the mean values were calculated. The reproducibility was ±0.2‰ for *δ*^13^C and ±0.3‰ for *δ*^15^N.

### 4.5. Statistical Analyses

The statistical calculations of the linear mixed models and data presentation were performed using R version 4.0.4 (R Foundation for Statistical Computing, Vienna, Austria) [61] and the software packages lmerTest (version 3.1.3.), emmeans (version 1.7.0.), factoextra (version 1.0.7.) and ggplot2 (version 3.3.5.). Due to the incomplete design and lack of randomization of treatments (N rates and agronomic management practices) in the long-term field experiment, statistical calculations for this oat study were performed separately for Trial 1 and Trial 2. Normal distribution and homogeneity of variance of residuals were tested using diagnostic plots and Levene’s test. Where necessary, data were transformed using the log10 transformation before, and the back-transformed data are presented in the figures. The statistical model for comparing treatments on agronomic performance traits (grain and straw yield, HI, TKW, plant height and lodging) used the following factors: T, treatment; R, complete replication; and Y, year. The F effects were considered fixed factors here, while Y, T × Y, and R × Y were modeled as random effects. The year was treated as a random factor due to the unpredictability of seasonal weather. The model used for statistical analysis of agronomic performance traits was as follows: MODEL1 = T: Y + Y × R + Y × T + T × Y × R. The residual error term was T × Y × R. The model was applied to 2015–2020 agronomic trait data. The statistical model used to compare treatments on macro- and microelement composition and stable isotope ratios were different. Here, T and Y’s effects were considered fixed effects, while the T × Y and R × Y effects were modeled as random effects. The model used for the statistical analysis of macro- and microelements and stable isotopes was as follows: MODEL2 = T + Y: Y × R + Y × T + T × Y × R. The residual error term was T × Y × R. The model was applied to the multi-element and stable isotope composition data of air-dried oat grains from two consecutive years (2016–2017). The Satterth–Waite approximation of the degrees of freedom was used in the statistical models. When ANOVA indicated statistical differences, Tukey’s post hoc test for multiple comparisons was used (*p* ≤ 0.05). Separate PCA was performed for agronomic trait data (2015–2020) and elemental and stable isotope composition data (2016–2017).

## 5. Conclusions

This study summarizes the evaluation of different cultivation practices on the agronomic performance of oats and the elemental composition and stable isotope ratio of N and C in oat grains. Comparing agronomic management practices of the long-term experiment (i.e., control without organic amendment, incorporation of manure every third year and incorporation of crop residues/cover crop) showed no differences while applying synthetic N (rates of 0, 55, 110 and 165 kg/ha) improved the oat grain and straw yield and increased susceptibility to lodging. Intensification of N fertilization was only possible up to a rate of 110 kg/ha. Application of synthetic N reduced Ca, Zn, Ti, Si, and Br content and increased Fe and Rb content in oat grains. The reduction in element content in grains with the intensification of oat cultivation is attributed to a dilution effect, where a proportional increase in element content does not accompany improved dry matter production at higher N rates. Further, based on the elemental composition and the total amount of elements accumulated in oat grains, no evidence of soil microelement depletion was observed in the 23rd and 24th years of intensive cultivation compared to low intensity cultivation in the long-term field experiment. Synthetic N application also reduced mean *δ*^15^N values in oat grains and increased *δ*^13^C values. Based on the *δ*^15^N values, only a difference between the values with and without synthetic N application above the synthetic N rate of 55 kg/ha was detected, while none of the stable isotopes indicated a difference between the management practices of the long-term field experiment. To the best of the authors’ knowledge, these are the first published results for multi-elemental composition and *δ*^15^N or *δ*^13^C values of oats and their changes as a function of N rate and agronomic management practice. 

## Figures and Tables

**Table 1:** Summary data from the linear mixed model ANOVA for agronomic performance traits of oats during 2015–2020.

Parameter	Grain Yield		Straw Yield		HI		Plant Height		TKW		Lodging	
Design	Trial 1	Trial 2	Trial 1	Trial 2	Trial 1	Trial 2	Trial 1	Trial 2	Trial 1	Trial 2	Trial 1	Trial 2
*p* value (treatment)	<0.001	<0.001	<0.001	<0.001	0.482	0.245	<0.001	<0.001	<0.001	0.036	<0.001	<0.001
R^2^ (marg.)	0.59	0.45	0.71	0.55	0.03	0.05	0.60	0.60	0.34	0.04	0.85	0.92
R^2^ (cond.)	0.93	0.89	0.88	0.84	0.80	0.80	0.82	0.85	0.89	0.78	0.92	0.97
AIC	1301.7	1302.5	1384.1	1384.9	560.2	559.4	600.8	574.8	354.4	379.2	144.4	132.4
BIC	1324.2	1325.0	1406.6	1404.4	582.7	581.9	623.3	597.3	376.9	401.7	166.9	154.9
Log likelihood	−641.8	−642.2	−683.0	−681.9	−271.1	−270.7	−291.4	−278.4	−168.2	−180.7	−63.2	−57.2

HI, harvest index; TKW, thousand kernel weight; marg., marginal; cond., conditional; AIC, Akaike information criterion; BIC, Bayesian information criterion. Straw yield data in both trials and TKW data in Trial 2 were normalized using the log10 transformation.

**Table 2:** Summary data of the linear mixed model ANOVA for macroelements in oat grains produced during 2016–2017.

Parameter	Si		Ca		K		P		S		Cl	
Design	Trial 1	Trial 2	Trial 1	Trial 2	Trial 1	Trial 2	Trial 1	Trial 2	Trial 1	Trial 2	Trial 1	Trial 2
*p* value (year)	0.591	0.002	0.266	<0.001	0.948	0.635	0.201	0.373	0.408	0.758	0.807	0.174
*p* value (treatment)	0.215	0.002	0.045	0.002	0.193	0.237	0.928	0.791	0.142	0.980	0.998	0.987
R^2^ (marg.)	0.30	0.55	0.31	0.58	0.27	0.18	0.17	0.11	0.22	0.03	0.02	0.18
R^2^ (cond.)	0.40	0.55	0.37	0.59	0.40	0.18	0.51	0.27	0.23	0.30	0.69	0.66
AIC	7.7	−8.2	−26.2	−32.4	59.5	70.8	61.9	65.0	−6.0	12.5	−61.8	−49.1
BIC	18.9	4.5	−13.6	−19.8	72.1	82.0	74.6	76.2	−5.2	25.1	−49.2	−37.9
Log likelihood	4.1	13.1	22.1	25.2	−20.7	−27.4	−22.0	−24.5	11.0	2.8	39.9	32.6

Marg., marginal; cond., conditional; AIC, Akaike information criterion; BIC, Bayesian information criterion. Data on the content of Si was normalized in both trials using the log10 transformation.

**Table 3:** Summary data of the linear mixed model ANOVA for microelements in oat grains produced during 2016–2017.

Parameter	Zn		Fe		Ti		Br		Rb		Sr	
Design	Trial 1	Trial 2	Trial 1	Trial 2	Trial 1	Trial 2	Trial 1	Trial 2	Trial 1	Trial 2	Trial 1	Trial 2
*p* value (year)	0.069	0.156	0.027	<0.001	0.728	0.328	0.607	0.070	0.042	0.011	0.009	0.008
*p* value (treatment)	0.011	<0.001	0.044	0.275	0.053	0.003	0.142	0.158	0.255	0.242	0.989	0.638
R^2^ (marg.)	0.74	0.75	0.50	0.43	0.66	0.80	0.53	0.58	0.48	0.65	0.58	0.54
R^2^ (cond.)	0.77	0.79	0.57	0.43	0.89	0.83	0.87	0.86	0.63	0.76	0.73	0.60
AIC	134.3	131.7	223.0	211.7	185.2	177.5	−8.7	−3.6	95.0	−2.9	−4.2	2.3
BIC	146.9	144.4	235.6	224.3	197.8	190.1	4.0	9.0	107.6	9.69	8.4	15.0
Log likelihood	−58.1	−56.9	−102.5	−96.9	−83.6	−79.8	13.3	10.8	−38.5	10.5	11.1	7.8

Marg., marginal; cond., conditional; AIC, Akaike information criterion; BIC, Bayesian information criterion. The content of Br and Sr (Trial 1 and 2) and Rb (Trial 2) were normalized using log10 transformation.

**Table 4:** Summary data obtained of the linear mixed model ANOVA for stable isotope ratios of N and C in oat grains produced during 2016–2017.

Parameter	*δ*^15^N		*δ*^13^C	
Design	Trial 1	Trial 2	Trial 1	Trial 2
*p* value (year)	0.194	0.546	<0.001	<0.001
*p* value (treatment)	0.040	0.039	<0.001	0.006
R^2^ (marg.)	0.64	0.60	0.87	0.95
R^2^ (cond.)	0.94	0.90	0.88	0.96
AIC	78.6	81.9	30.4	10.1
BIC	91.3	94.5	43.0	22.7
Log likelihood	−30.3	−31.9	−6.2	4.0

Marg., marginal; cond., conditional; AIC, Akaike information criterion; BIC, Bayesian information criterion.

**Table 5:** Treatment combinations of synthetic N rate and agronomic management practices of the long-term experiment used for oats in this study.

Trial	Design	Treatment		Application of Synthetic N (kg/ha)		
		Management Practice	Synthetic N Fertilization (kg/ha)	BBCH 21/22	BBCH 31/32	BBCH 45/50
1	No.org–N0	na	na	na	na	na
	Manure–N0	Manure	na	na	na	na
	Manure–N1	Manure	55	55	na	na
	Manure–N2	Manure	110	55	55	na
	Manure–N3	Manure	165	70	70	25
2	No.org–N3	na	165	70	70	25
	Straw–N0	Straw	na	na	na	na
	Straw–N1	Straw	55	55	na	na
	Straw–N2	Straw	110	55	55	na
	Straw–N3	Straw	165	70	70	25

No.org, no organic amendment applied and aboveground biomass of crops removed; Manure, manure applied every third year before sowing of maize at the rate of 30 t/ha and aboveground biomass of crops removed; Straw, aboveground residues of crops in the rotation incorporated in the soil and cover crop sown every third year after oats; N0, N1, N2, N3, synthetic N added at rates 0, 55, 110 and 165 kg/ha; na, not applicable; BBCH, phenological development stages of plants according to the BBCH scale.

**Table 6:** Mean daily air temperature and cumulative monthly precipitation from sowing to harvest in the 2015–2020 growing seasons.

Year	2015	2016	2017	2018	2019	2020	Mean 2015–2020
Temperature (°C)							
March	6.1	6.3	8.3	3.6	7.3	6.0	6.3
April	10.2	11.2	10.6	13.6	10.6	11.1	11.2
May	15.6	14.0	15.8	16.9	12.0	14.0	14.7
June	18.9	18.6	20.4	19.4	21.9	18.2	19.6
July	22.7	21.4	21.2	20.5	21.4	20.3	21.3
August	20.7	19.2	21.4	21.2	21.3	21.0	20.8
Mean	15.7	15.7	16.3	15.9	15.8	15.1	15.6
Precipitation (mm)							
March	93.0	94.4	41.4	128.6	51.0	101.2	84.9
April	45.2	28.2	172.2	90.4	101.6	21.6	76.5
May	130.8	173.0	44.2	108.8	217.8	117.6	132.0
June	36.0	181.8	148.8	86.2	57.6	169.2	113.3
July	110.0	81.6	90.6	195.6	127.0	188.7	132.3
August	74.6	99.4	43.8	149.2	136.8	120.0	104.0
Sum	489.6	658.4	541.0	758.8	691.8	718.3	643.0
Solar radiation (W/m^2^)							
March	225.3	171.9	263.5	174.0	246.7	119.8	268.8
April	303.6	272.3	280.2	314.2	239.6	202.9	264.4
May	280.0	272.0	334.6	303.5	216.4	179.6	314.1
June	338.3	301.7	336.0	320.3	372.6	215.7	327.2
July	339.8	333.4	364.5	316.4	345.9	262.9	308.1
August	317.0	335.1	357.8	334.7	314.6	189.2	280.4
Sum	1804.0	1686.4	1936.6	1763.1	1735.8	1170.1	1763.0

## Data Availability

The data that support the findings of this study are available on request from the corresponding author. The data are not publicly available due to current project restrictions.
